# A model for community health service development in depressed rural areas in China

**DOI:** 10.1186/1472-6963-12-465

**Published:** 2012-12-17

**Authors:** Yuan Zhaokang, Liu Yuxi, Liu Yong, Xiao Yunchang, Guo Yuanjun, Mark Harris

**Affiliations:** 1 School of Public Health, Nanchang University, 461 Baiyi Road, Nanchang, Jiangxi, 330006, China; 2 Rural Health Office of Jiangxi Provincial Health Bureau, 6 Xi’er Road in courtyard of Jiangxi Province government, Nanchang, Jiangxi, 330000, China; 3 Health Bureau of Chongyi County, 80 West Street, Chongyi County, Jiangxi, 341300, China; 4 School of Public Health and Community Medicine, University of New South Wales, Sydney, NWS, 2052, Australia

**Keywords:** Primary health care, Community health services, Rural health, Economically depressed, Public health

## Abstract

**Background:**

To introduce a model of community health service organization (as implemented in urban areas) to less developed rural areas in China and evaluate the impact of this model on health care utilization.

**Methods:**

The intervention involved developing leadership at county level, training rural health practitioners, providing clinical management guidelines and standards, encouraging clinic improvements and providing access to subsidies for public health work. We chose 7 townships and 49 administrative villages in Chongyi County as the intervention sites; 3 townships and 9 administrative villages in Luxi County as the comparison sites. Officers from county health bureaus and postgraduates from School of Public Health, Nanchang University visited each township hospital and village clinic in field together and made observations and interviewed clinic staff.

**Results:**

There was little change in health facilities or workforce in the two areas. However, there was an increase in the use of public health services at township and village level in the intervention sites in Chongyi. In these, the proportion of clinics which had developed a child health (under the age of 3) management system, maternal postpartum visit and chronic disease management increased from 53%, 51% and 47% to 78%, 73%, and 71% respectively. There was no significant change in the comparison sites.

**Conclusions:**

The trial demonstrated that it was feasible to implement a model of community health service delivery that was adapted to depressed rural areas because it required little organizational change, additional funding or personnel. The model had a positive impact on the provision of public health programs, a finding which has implications for efforts to improve access to primary health care in rural China.

## Background

800 million people live in rural China [1]. Due to historical and economic factors, governments have given less attention to health service development in rural than in urban areas [2], resulting in a relatively weak rural health system [3]. Economically depressed rural areas are located in the Chinese central and western regions. In these regions the level of the funding available for primary health care is less than in urban areas and coastal regions and there is a widening gap in access to health care [4,5]. Rural doctors tend to give more focus to management of acute clinical problems than to public health or preventive medicine [6]. This contributes to the poor health status of the rural population. In 2009, infant mortality and maternal mortality were much higher in rural than urban areas (17.0 ‰ vs 6.2 ‰, and 34.0 vs 26.6 per100,000 respectively) [7]. There is an urgent need to improve the quality of health service in rural areas.

In China, community health services include the "Six in One" functions carried out in primary health care institutions, including preventive health care, basic medical care, health education, rehabilitation and family planning, most of which are related to public health programs. The introduction of this model has been complemented by the introduction of the cooperative medical insurance for rural China [8]. The implementation of the community health services model in the rural areas has the potential to change the concepts and service models of basic medical personnel from a passive to active approach, putting greater emphasis on disease prevention and control and the accessibility of health services.

In recent years, in the economically developed areas in China (e.g. around Beijing, Shanghai, Zhejiang), rural community health services (CHSs) have been established. These have required significant government investment. In 2008 the provincial government provided special funding for public health services in more developed rural areas (e.g. over 15 Yuan per capita in Zhejiang) [9]. These initiatives have achieved good results. For example, rural hospitals and clinics in Zhejiang have been converted to Community Health Service Centres (CHSC) and Community Health Service Stations (CHSS) [10]. In 2008, infant mortality and maternal mortality in Shanghai were lower than the national level [11,12]. To date there have been no reports of studies evaluating the application of the CHS model in economically depressed rural areas. This paper describes the implementation of a form of the CHS model adapted to an economically depressed rural area and its impact on service provision.

## Methods

### Context

Chongyi, the intervention site, is a small county in a remote mountain area. It has a population of 203,438, of which 165,214 work in agriculture. The fiscal revenue of local government was 307,700,000 Yuan and net average income of farmers for the whole year was 3,406 Yuan per capita in 2008, which is below the 2008 average per capita net income of rural residents in China (4,761Yuan). The economic conditions of Chongyi County are representative of depressed rural areas. There is a fairly sound three-level health service system (village, township and county). Luxi (the comparison site) is also a small county in mountain area adjacent to Chongyi in Jiangxi province. It has a population of 286,300 and an agricultural population of 227,733. The fiscal revenue of local government was 378,000,000 Yuan in 2008. The per capita annual income was 5047 Yuan.

Before the implementation of the project, there were no appraisal standards for township hospitals and village clinics. Rural doctors were not aware of the importance of proactive care, passively relying on patients initiating contact with the doctor. They did not provide planned visits for patients with chronic diseases such as hypertension, diabetes and special populations such as maternal, children and the elderly.

### Study population and sample

Seven townships were randomly sampled from the 16 townships in Chongyi County. These 7 townships included 49 administrative villages. Three townships were randomly selected from a total of 10 townships in Luxi County. In each township, 3 administrative villages were selected – a total of 9 administrative villages.

### Data collection

Officers from county health bureau and postgraduates from School of Public Health, Nanchang University interviewed the staff in each township hospital and village clinic together, using a questionnaire. The questions were adapted from the national health resources and medical service survey system [13]. They included questions about health service buildings and facilities, equipment, service functions and staff numbers, as well as types of service carried out. According to “Jiangxi township hospitals construction standards”, the following services should be conducted: - disease prevention and health care; basic medical treatment; family planning; and health management. Thus respondents were asked to indicate if the following specific areas were provided:

Public health functions including:

○ Establishment of family health records

○ Immunization

○ Infectious disease control

○ Chronic non-communicable diseases prevention and treatment (eg hypertension)

○ Maternal and child health care

○ Aged care and rehabilitation

○ Family planning counseling

○ Health education

Clinical care including the provision of

○ Routine physical check

○ Laboratory check

○ TCM basic technology

○ Gynecological routine inspection

○ Referral in

○ Referral out

○ Home visit

○ Home care

○ Home sickbed

This questionnaire has been used in previous field studies by the School of Public Health.

### Statistical analysis

Univariate descriptive analysis including Pearson Chi Square was conducted using the Statistical Package for Social Sciences (SPSS 18).

### Ethics

The study was approved by the Human Research Ethics Committee of Nanchang University.

### Intervention model

1. The development of the model

CHS policies and reports from urban and more economically advanced rural areas (e.g. Zhejiang, Jiangsu) were reviewed and joint visits were conducted to examine the model of rural CHS in Zhejiang Province. Analysis of policy and a literature review provided the foundation from which we developed the initial Chongyi County CHS Model. This was then refined by a multi-stage process of iterative feedback and revision in consultation with the county government in 2009. Participants in this consultation came from the relevant branches of the health, social security, civil administration and financial administration in the county. The model was continually revised in response to the multiple stakeholder consultations, using a method similar to that used to develop performance measurement for CHSs [14]. Participants discussed the CHS progress, achievements and problems, and were asked a series of open-ended questions. Examples of questions included: “Is the rural CHS in Chongyi sustainable?” and “Should there be any immediate outcomes of services provided by CHS facilities?” A record was made of the consultation meetings. This was analysed thematically and summarized. This was then provided to the participants for comment. Finally they were used to refine model, revise regulations, standards and incentive polices.

2. Main features of the model

The model introduced the concept and organization of CHSs and aimed to strengthen the capacity of health-care institutions at township and village levels. At village level, public health services to be introduced included special clinics for maternal health, child health, immunization and chronic disease; health education using health materials; outreach into people’s homes; establishing health records for the whole population. At township level, public health and clinics were introduced along with development of a role in health management, supervision and education of doctors in Village Health Service Stations. At all these levels, a single electronic health record system was developed based on a card that provided access to health records and health insurance. The record system was accessible via secure Internet link at village, township and county level.

3. Implementation of the model

The steps involved in implementation included:

(1) Promoting involvement of the county government in the development of primary health care(PHC).

Engagement of rural government officials was achieved through seminars, individual interviews, joint review of relevant documents, and joint visits to examine the model of rural CHSs in Zhejiang province. The local government established a group to lead rural CHS work, with leadership from the relevant branches of the health, social security, civil administration and financial administrations. This helped to integrate PHC into the government’s work plan and to develop policies to promote PHC.

(2) Improving the skills of PHC practitioners.

Training aimed to improve the individual skills of primary health care workers. The project group conducted a 1-week training course for 40 health managers, 160 doctors and 40 nurses. This included both theoretical education and field-based training. Theoretical training included social medicine, community medicine, general medicine, community health service, community nursing, and health management. Chongyi County also sent 20 doctors from township hospitals in two batches to participate in transition training (6 months GP training) conducted by the provincial health department. The immediate impact of the training on their knowledge, attitudes and stated practice was assessed using a questionnaire before and after the training. These were analyzed to evaluate the quality of training.

(3) Improving management processes in PHC

Chongyi County health management regulations were revised and the Chongyi County CHS standards were drafted. The major standards were based on those in urban areas, adapted for conditions in depressed rural areas. They were discussed and revised three times by government officials (provincial, municipal, county and township) and the experts from the university.

(4) Improving access to PHC

To encourage medical workers at village and township levels to carry out effective PHC, incentive policies for rural CHSs were introduced in Chongyi County. The policies included funding for repair or refurbishment of clinic premises and some additional equipment (computer, examination bed, simple test equipment). By establishing these township and village health facilities as new rural cooperative medical care institutions, the rural doctors were able to access subsidies for public health service work (e.g. home visits, immunizations, patient education).

During the study, the management systems of rural community health services were improved:

In the business management system, planning for immunizations, emergency treatment for public health emergencies, women health care, child health care, geriatric care, community-based rehabilitation, health education, family planning and technical guidance was established or enhanced.

In technical norms and work systems, procedures for emergency first aid, outpatient work, and the responsibilities for initial diagnosis were improved.

In the management systems for rural community health service stations, standards for the behavior of community health service staff, the responsibilities of general practitioners, the responsibilities of community nursing, elder care work, prescription management and disinfection management were established. Also the technical specifications of treatment rooms were modified.

Continuing education and technical appraisal, quality improvement supervision, accident prevention, medical waste disposal and pharmacy staff education were improved.

In the comparison sites in Luxi County, these interventions were not provided and township and village health facilities provided usual care.

### Funding

The intervention was resourced largely by the Health Bureau of Chongyi County. This included funding for repair and refurbishment of clinical premises and provision of some additional equipment. It also included the time of county staff involved in training and supervision and travel costs.

The training of health administrators, township and village doctors and community nurses was funded by project funds the China-Australia Health and HIV/AIDS Facility. The project funds also included travel and accommodation for evaluation field staff and visits by the Australian consultant to Chongyi.

Incentives for rural doctors’ work were provided through the rural cooperative health insurance scheme and funding for public health services.

## Results

There was little difference in investment in health facilities or in the workforce in the two areas during the evaluation period. Training cost RBM100 Yuan / worker per day (a total cost of RMB168,000 Yuan for 240 health-care workers) which translates to an average cost of 0.84 Yuan per rural resident. The costs of supervisory visits to each clinic was about RMB150 Yuan per visit and the cost of implementing revised management regulations and local incentive polices were low and absorbed with routine activities.

### Township level

The “The state's basic public health service standards” [15] require the following public health services to be provided: Establish family health archives; Health education; Inoculation; Infectious disease report and treatment; Chronic non-epidemic diseases system management; Child health system management; Maternal system management; Elderly system management; Maternal system management; Rehabilitation service; Health management. In 2009 in Chongyi there were deficiencies in child health, elderly care. By 2010 these standards were all met, except in one institution that did not conduct family planning counselling and rehabilitation. In Luxi, most hospitals did not provide public health services and this did not change (Table 1).

**Table 1:** Number of township hospitals carrying out the public health service programs in two counties in 2009 and 2010

**Project name**	**Chongyi(n=7)**		**Luxi (n=3)**	
	**2009**	**2010**	**2009**	**2010**
Establish family health archives	7	7	0	0
Inoculation	7	7	0	0
Infectious disease control	7	7	1	1
Chronic non-epidemic diseases management	7	7	0	1
Maternal system management	7	7	1	1
Child health system management	6	7	1	1
Elderly system management	4	7	0	0
Family planning counselling	3	6	1	1
Health education	7	7	1	1
Rehabilitation service	3	6	2	2
Health management	7	7	3	3

In Chongyi, in 2009, there were deficiencies in arrangements for the transfer in or referral out of patients to other hospitals or home care. In 2010, these had improved except for transfer in of patients to the service. There was no change in the provision of these services in Luxi (see Table 2).

**Table 2:** Number of institutions carrying out basic medical services in two counties in 2009 and 2010

**Project name**	**Chongyi (n=7)**		**Luxi (n=3)**	
	**2009**	**2010**	**2009**	**2010**
Routine physical check	7	7	3	3
Laboratory check	7	7	3	3
Traditional Chinese Medicine	4	6	1	1
Gynaecological routine inspection	7	7	3	3
Referral in	3	3	2	2
Referral out	6	7	2	2
Home visit	6	7	2	2
Home care	3	4	0	0
Home sickbed	5	6	0	0

### Village level

A number of public health services increased at village level in Chongyi but not in Luxi. In Chongyi, the proportion of clinics which had implemented the child health management system (for children under 3 years), maternal postpartum visits and chronic disease management increased from 53%, 51% and 47% to 78%, 73%, and 71% respectively. Other services did not change, and there were no significant changes in Luxi (Table 3).

**Table 3:** Health service agency number of village clinics in two counties in 2009 and 2010

**Service item**	**Chongyi (%)**		**χ**^**2**^	**P**	**Luxi (%)**		P*
	**2009 (n=49)**	**2010 (n=45)**			**2009 (n=9)**	**2010 (n=8)**	
Children (under the age of 3) system management	26 (53.1)	35 (77.8)	6.290	0.012	3 (33.3)	3 (37.5)	1.000
Maternal postpartum visit	25 (51.2)	33 (73.3)	4.942	0.026	4 (44.4)	4 (50.0)	1.000
Patient registries	49 (100.0)	45 (100.0)	-	1.000	8 (88.9)	8 (100.0)	1.000
Planed immunization	48 (98.0)	45 (100.0)	-	1.000	8 (88.9)	7 (87.5)	1.000
Surveillance of communicable diseases	49 (100.0)	45 (100.0)	-	1.000	8 (88.9)	8 (100.0)	1.000
Chinese medicine diagnosis	45 (91.8)	44 (97.8)	-	0.364	8 (88.9)	8 (100.0)	1.000
TB management	31 (63.3)	29 (64.4)	0.014	0.905	6 (66.7)	7 (87.5)	0.576
Establish health archive	30 (61.2)	34 (75.6)	2.217	0.136	2 (22.2)	2 (25.0)	1.000
Chronic disease management	23 (46.9)	32 (71.1)	5.646	0.017	8 (88.9)	4 (50.0)	0.131
Health education	48 (98.0)	45 (100.0)	-	1.000	8 (88.9)	4 (50.0)	0.131

*Fisher Precision probability.

## Discussion

In recent years, CHSs have been established in economically developed rural areas. Resource limitations have prevented application of this model of CHS delivery in economically depressed rural areas. The consultations conducted as part of the development of the model demonstrated a need for policies for rural community health services which are not only consistent with the rural reality, but also conducive the development of rural community health services. The development of rural community health services requires an integrated approach to standards and training at the village, township and county levels that is consistent with national policy. Evaluation of this model needs to consider the practicalities of implementation and sustainability over the long term.

This study demonstrated that implementation of a modified CHS model was feasible and had a positive impact on the public health services provided at village and township level. This model involved both public and private health care providers (as staff in town ship hospitals were employed by government, whereas most village doctors were self employed). It required both appropriate incentive policies and support to increase the motivation of the village doctors and appropriate rules and regulations governing the behavior of village doctors’ community health service provision. Its implementation involved developing leadership to provide policy and support for implementation; training rural health practitioners to change their conceptualization and pattern of service provision; setting standards to ensure institutionalization of the change in health service delivery; and developing encouraging supervision to promote change in the service model of rural health practitioners.

The model required active engagement of county level officials. Prior to the implementation of the project, a good base had been developed for rural health work in Chongyi County. It had won several awards as an advanced county in rural health work, and this laid a good foundation for the introduction of rural community health service model. The local government was highly concerned about the health of their residents and sought to improve this through the implementation of the project.

The commitment of local leadership further developed through discussions and visits to observe and study the rural community health services in Zhejiang Provice and guidance from foreign and domestic experts during their visits to Chongyi.

This model is suitable for other economically depressed rural areas in China. County governments can introduce this model by modifying their existing system with limited funding. The project required limited investment by government, and did not involve the transformation of institutions and increased manpower. The costs of training and supervisory visits were comparatively low. The model was successfully implemented and achieved changes in the implementation of public health services and programs targeting pregnant women, children, and patients with chronic disease, especially at the village level. This is important, given the prior emphasis on reactive management of clinical problems by doctors at this level [7].

In this study the School of Public Health, Nanchang University had an important role across all phases of the development and implementation of the model. It had previously developed a relationship with the local Health Bureau. Its role included negotiation with local health officials on the design, implementation and local resourcing of the project, developing the project plan, working with local leadership to develop policies, incentives and regulations. The School also participated in the implementation of the model through onsite training and visits to facilities. In implementing the model in other areas, these roles may be provided by Schools of Public Health or by agencies such as the Centres for Disease Control.

There are a number of limitations to this study. It was conducted in rural areas in one province, and application of the findings to other areas needs to be done with caution. The design was quasi-experimental and it possible that differences between the study sites might be explained by other factors. More analysis and evaluation, including cost-benefit analysis, are needed to further inform the future development of this model. The most significant change was in the number of services provided in village clinics in Chongyi. There is a possibility of reporting bias. However this was evaluated by field visits, routine reporting and surveys which were validated both by investigators from the University and local Chongyi Health Bureau. It was also confirmed in field visits to a sample of village clinics by the national and international consultants.

## Conclusion

The CHS model was adapted by an iterative process of consultation to rural health services in a depressed county and was implemented effectively. The model engaged services at village and township level and included both public and private providers. It was feasible because it did not require major organizational change, additional funding or personnel. It could be implemented over 2 years because of the strong engagement of the county government. The changes were associated with improvements in the provision of some public health services in the pilot area. While further research is needed, application of the model in other depressed rural areas should be explored.

## Competing interests

Financial competing interests: None of the authors have financial competing interests to declare.

## Authors' contributions

YZ from the School of Public Health, Nanchang University was responsible for the project, including project design, site implementation and supervision. He led the authorship of this paper. LY and LY were involved in the evaluation data collection and analysis and critically reviewed and commented the draft paper. XY coordinated the project support in Chongyi and critically reviewed and commented the draft paper. GY contributed to the implementation of the project intervention in Chongyi and critically reviewed and commented on the draft paper. MH was advisor to the project during the design, implementation and evaluation stages. He visited Chongyi and provided comment and advice and during visits of the researchers to Australia discussed the analysis and writing. He reviewed and edited the paper. All authors read and approved the final manuscript.

## Pre-publication history

The pre-publication history for this paper can be accessed here:

http://www.biomedcentral.com/1472-6963/12/465/prepub
